# Predictive value of prenatal screening markers combined with serum placental growth factor in early pregnancy for preeclampsia

**DOI:** 10.12669/pjms.41.2.9794

**Published:** 2025-02

**Authors:** Zitan Shu, Weiwei Wang

**Affiliations:** 1 Zitan Shu, Department of Laboratory Medicine, Jingmen Central Hospital, Jingmen 448001, Hubei, China; 2 Weiwei Wang, Department of Operating Room, Jingmen Central Hospital, Jingmen 448001, Hubei, China

**Keywords:** Early pregnancy, Markers, Serum placental growth factor, Preeclampsia, Predictive value

## Abstract

**Objective:**

To observe the predictive value of prenatal screening markers combined with serum placental growth factor(PLGF) in early pregnancy for preeclampsia(PE).

**Methods:**

This was a prospective study. A total of 369 pregnant women undergoing early pregnancy examinations were selected at Jingmen Central Hospital from August 2024 to January 2025 and divided into the PE group(n=43) and the normal group(n=326) according to the presence of PE during the follow-up. The levels of prenatal screening markers alpha-fetoprotein(AFP), serum PLGF, β-human chorionic gonadotropin(β-hCG) and pregnancy-associated plasma protein-A(PAPP-A) were compared between the two groups.

**Results:**

There were 43 patients experiencing PE, with an incidence of 11.65%. The levels of PLGF, β-hCG and PAPP-A were significantly decreased in the PE group compared with those in the normal group, and the differences between the groups were statistically significant (all P<0.05). Logistic regression analysis showed that increased prenatal screening markers AFP, serum PLGF, HCG and PAPP-A were independent risk factors for PE, with statistically significant differences between the groups (all P<0.05). Finally, the results of ROC curve analysis showed that the AUCs of AFP, PLGF, β-hCG and PAPP-A were 0.618, 0.645, 0.690, and 0.645, respectively, and the AUC of combined prediction was 0.825, which was significantly increased compared with that of each marker alone, with statistically significant differences(P<0.05).

**Conclusion:**

The development of PE in pregnancy is closely related to the levels of AFP, PLGF, β-hCG and PAPP-A. The predictive efficiency of combined detection of AFP, PLGF, β-hCG and PAPP-A for PE in pregnancy significantly increases.

**Project Title:** sFlt-1/PlGF based combined early pregnancy prenatal screening marker Nursing Strategies and Application in Preeclampsia Risk Assessment, Project Number: 2024YFYB111, Year: 2024.

## INTRODUCTION

Preeclampsia (PE) is a common disease during pregnancy,^1^ and usually occurs after 20 weeks of pregnancy, with the main clinical features of hypertension and proteinuria. The pathogenesis and etiology of PE are complicated and not fully established. This disease originates from the placenta and may involve various organs and systems. Placental abruption, heart failure, and disseminated intravascular coagulation may occur in severe cases, which seriously threatens the life and health of mothers and infants.^2^-^4^ Although a series of studies on independent risk factors for PE have been conducted in recent years, more comprehensive prediction methods are still needed for the prediction and improvement of PE.^5^

The common screening method currently used in clinical practice is Down syndrome screening. The risks of neural tube defects, trisomy 18 syndrome and trisomy 12 syndrome are predicted by the testing results of serum markers in maternal peripheral blood as well as other factors such as the gestational week, gravidity, and body weight.^6^ Serum markers commonly used in clinical screening include AFP and β-hCG. Studies on PE have shown that the serum markers in these pregnant women are usually abnormal, which may indicate the occurrence of PE.^7^ PLGF is a member of the vascular endothelial growth factor family. Angiogenesis is an essential process during embryonic development, and PLGF can bind to cell growth factors to form heterodimers to inhibit or promote angiogenesis.^8^ PAPP-A is a glycoprotein produced by the placenta.

Studies have confirmed that abnormal expression of serum PAPP-A and PLGF in early pregnancy may affect the development of PE.^9^ However, the specificities of these markers for disease diagnosis are relatively low. On this basis, prenatal screening markers combined with serum PLGF in early pregnancy were selected to observe the predictive value of prenatal screening markers combined with serum placental growth factor (PLGF) in early pregnancy for preeclampsia (PE).

## METHODS

This was a prospective study. Three hundred and sixty-nine pregnant women undergoing early pregnancy examinations in Jingmen Central Hospital between August 2024 to January 2025 were selected and divided into the PE group(n=43) and the normal group(n=326) according to the presence of PE during the follow-up. The pregnant women with an average age of 27.22±3.31 years old (range 22-35), an average gestational age of 27.33±5.33 weeks (range 10-13 weeks), and an average body weight of 63.02±1.92 kg (range 55-70 kg), were retrieved from electronic medical record systems.

### Ethical approval:

The study was approved by the Institutional Ethics Committee of Jingmen Central Hospital (No.: 2024YFYB111; Date: August 7, 2024), and written informed consent was obtained from all participants.

### Inclusion criteria:

- With complete clinical data, and aged 18-35 years.
- With single intrauterine pregnancy.
- Who did not take hormone drugs recently.
- Who were willing to participate in maternal serum screening?
- Without mental disorders.
- Without serious organ diseases or infectious diseases.
- Obtained the informed consent of the family.

### Exclusion criteria:

- Twin or multiple intrauterine pregnancies.
- Malignant tumors or organ dysfunctions.
- Chronic diseases such as hypertension and diabetes.
- Chromosomal abnormalities diagnosed by relevant tests.
- Severe blood system diseases (acute leukemia, malignant lymphoma, multiple myeloma, etc).

### Methods:

Peripheral five ml venous blood was collected in all cases under fasting condition in the morning from the subjects at 10-13 weeks of gestation, serum was isolated and stored at -20°C. The levels of serum AFP, β-HCG, PLGF and PAPP-A were determined by electrochemiluminescence using the UniCel DxI800 immunoassay system (Beckman Coulter, USA) and reagents from Roche. The tests were conducted strictly following the corresponding instructions.

Preeclampsia (PE) in these pregnant women was diagnosed according to the diagnostic criteria in the Obstetrics and Gynecology, 8^th^ edition^10^, and divided into early onset (<34 weeks of gestation) and late onset (≥34 weeks of gestation) PE according to the time of onset.

### Outcome Measures:

- The general data of pregnant women were compared between the PE group and the normal group, including age, gestational age, body weight and gravidity;
- The levels of AFP, β-HCG, PLGF and PAPP-A were compared between the two groups;
- Risk factors were analyzed for women in the PE group using logistic regression analysis;
- The predictive value of each marker for PE in pregnancy was analyzed using the ROC curve.

### Statistical Analysis:

The levels of serum markers in pregnant women of the two groups were analyzed using SPSS 24.0 software. The incidence was presented as n (%), the measurement and enumeration data were presented as (*χ̅*±*S*) and independent samples t test was used for comparison. The Chi square test was used for enumeration data. Differences with a p<0.05 were considered statistically significant. The effect of each factor on the development of PE was analyzed using the univariate and logistic binary regression analysis, and differences with a p<0.05 were considered statistically significant. The predictive value of risk factors for PE was evaluated via receiver operating characteristic curve (ROC) analysis using MedCalc 19.0 software, and area under curve (AUC) >0.5 was used as the cutoff value for the presence of predictive values, with AUC>0.9 indicating a high diagnostic value.

## RESULTS

A total of 369 pregnant women undergoing early pregnancy screening were included in the present study, with an average age of 27.22±3.31 years old (range 22-35), an average gestational age of 9.88±1.93 weeks, and an average body weight of 63.02±1.92 kg (range 55-70 kg). According to the testing results during follow-up, PE was diagnosed in 43 out of the 369 pregnant women included, and no PE was found in the remaining 326 women, with an incidence of 11.65%. Among the patients with PE, there were 10 cases of early onset PE and 33 cases of late onset PE. No statistically significant differences in general data were observed between the two groups of pregnant women (P>0.05) (Table-I).

**Table-I:** Comparison of general data between the two groups of pregnant women(*χ̅*±*s*).

Items	The normal group (n=326)	The PE group (n=43)	*t*	*p*
Maternal age(years)	27.61±4.22	28.14±3.16	0.785	0.434
Gestational age(weeks)	10.11±2.01	9.77±1.88	1.050	0294
Body weight(kg)	62.88±3.48	63.11±3.21	0.411	0.681
Gravidity(times)	1.55±0.61	1.48±0.87	0.669	0.504

The levels of prenatal screening markers AFP, PLGF, β-hCG and PAPP-A were compared between the two groups, and it was found that the level of AFP was significantly increased, and those of PLGF, β-hCG and PAPP-A were significantly decreased in the PE group compared with those in the normal group, respectively (all P<0.05) (Table-II).

**Table-II:** Comparison of the levels of prenatal screening markers and serum PLGF between the two groups(*χ̅*±*s*).

Items	The normal group (n=326)	The PE group (n=43)	*t*	*p*
AFP(ug/ml)	20.44±3.66	22.45±4.11	3.335	0.001
PLGF(U/ml)	89.36±14.99	80.89±13.77	3.514	0.000
β-hCG(ng/ml)	70.66±14.23	63.32±13.55	3.196	0.002
PAPP-A(IU/L)	30.28±5.44	27.08±4.88	3.667	0.000

Logistic regression analysis was conducted with the development of PE as a dependent variable, and AFP, PLGF, β-hCG and PAPP-A as independent variables, and the results showed that AFP, PLGF, β-hCG and PAPP-A were effective predictors for the development of PE (Table-III).

**Table-III:** Results of binary logistic regression analysis.

Factors	Regression coefficient	SE	Wald value	*P*	Correlation	95% CI	

						Upper limit	Lower limit
AFP	0.132	0.047	7.893	0.005	1.141	1.041	1.252
PLGF	-0.033	0.012	7.503	0.006	0.968	0.945	0.991
β-hCG	-0.037	0.012	9.428	0.002	0.964	0.941	0.987
PAPP-A	-0.071	0.035	4.083	0.043	0.931	0.869	0.998
constant	2.524	1.694	2.219	0.136	12.473		

**Fig.1:**
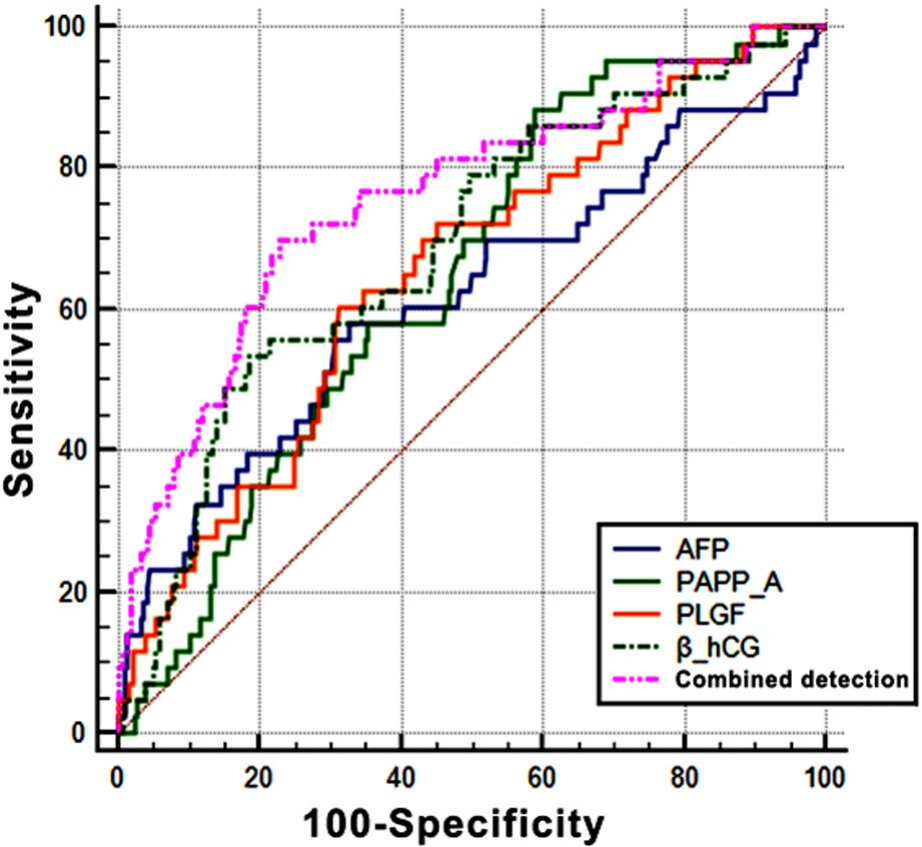
The area under ROC curve of AFP, PLGF, β-hCG and PAPP-A for predicting PE during pregnancy.

ROC curve analysis was performed with AFP, PLGF, β-hCG and PAPP-A as test variables, and the results showed that the AUCs of AFP, PLGF, β-hCG, and PAPP-A were 0.618, 0.645, 0.690, and 0.645, respectively, and the prediction probability of combined prediction was 0.825, which was significantly increased compared with that of each factor alone. The differences were statistically significant (P<0.05) (Table-IV and Fig.1).

**Table-IV:** Comparison of the area under the ROC curve of each factor.

Test variables	AUC	SD	p	95%CI	

				Upper limit	Lower limit
AFP	0.618	0.052	0.002	0.566	0.668
PLGF	0.645	0.044	0.017	0.603	0.702
β-hCG	0.690	0.044	0.018	0.640	0.737
PAPP-A	0.645	0.040	0.011	0.566	0.694
Combined detection	0.834	0.043	0.022	0.747	0.879

## DISCUSSION

The results of the present study showed that the level of AFP in the PE group was significantly increased compared with that in the normal group (P<0.05), indicating that an increased level of AFP may affect the development of PE. β-hCG is produced by fetal placental trophoblast cells. The expression level of β-hCG increases rapidly within the first eight weeks of gestation and remains stable at 20 weeks of gestation. At 16 weeks of gestation, the level of β-hCG decreases to one-fifth of its peak value, and this level will be maintained till the delivery period of the pregnancy. However, in a small number of pregnant women affected by Down syndrome, the level of β-hCG decreases slowly due to the poor development of placental function, and a relatively high level of β-hCG can be detected even during mid-pregnancy serum screening.^11^

It was found in the present study that the level of β-hCG in pregnant women was significantly decreased in the PE group compared with that in the normal group, and the difference was statistically significant(P<0.05), which was consistent with the findings of studies by Guo X et al.^12^, i.e., the level of serum β-hCG in pregnant women is related to placental abnormalities. Alpha-fetoprotein(AFP) is a monomeric glycoprotein present in the serum of pregnant women. As the main serum binding protein in the fetus, AFP involves the transportation of hormones and bilirubin. AFP is initially synthesized in the yolk sac, and later in the fetal gastrointestinal tract and liver.^13^ After synthesis, AFP is transported to maternal serum during fetal urine excretion through the fetal transmembrane or placenta.

The increased expression level of AFP in maternal serum during mid-pregnancy is closely related to fetal placental pathology and fetal structural abnormalities. Common fetal structural abnormalities include neural tube defects, abdominal wall defects, and gastrointestinal, skeletal, renal, and skin abnormalities. When abdominal wall defects or open neural tube defects occur in the fetus, the amount of amniotic fluid leaking from the fetus increases, and maternal serum concentration of AFP subsequently increases. In addition, the level of AFP level may increase when abnormal placental blood vessels are present. Studies^14^ have reported that the placental barrier is disrupted in fetuses with structural abnormalities, leading to placental ischemia or vascular bleeding and injury. Plasma protein-A(PAPP-A) is produced by placental trophoblast cells. It interacts with insulin growth factors to play an essential role in the growth and development of the fetus and placenta.^15^ Romero Infante XC et al.^16^ have shown that abnormal levels of serum PAPP-A during pregnancy are closely related to the development of PE.

The results of the present study showed that the level of PAPP-A in pregnant women was significantly decreased in the PE group compared with that in the normal group with a statistically significant difference(P<0.05), suggesting the predictive value of abnormal PAPP-A level for the development of PE. PLGF, a major cytokine in placentation, can promote the rapid formation of placental blood vessels, effectively enhance the proliferation and invasion of trophoblast cells in vivo, and increase the permeability of placental blood vessels.^17^ During the development and growth of the embryo in early pregnancy, the expression level of PLGF will increase accordingly to promote the angiogenesis of fetal placental branch blood vessels.^18^

Relevant studies^19^ have shown that the level of serum PLGF in normal pregnancy shows a peak shape, and the serum PLGF level during the first and second trimesters increases, and reaches a peak at 29-32 weeks of gestation, followed by a gradual decrease. The change in PLGF expression level can regulate cell infiltration and proliferation and affect the further development of villi. Meanwhile, low level of PLGF may impair the development of fetal blood vessels, and cause poor remodeling of fetal trophoblast cells and maternal spiral arteries, as well as placental hypoxia and ischemia, resulting in various placental defects and adverse pregnancy outcomes such as intrauterine fetal growth restriction and preterm birth.^20^,^21^

The results of the present study showed that the expression level of PLGF was significantly decreased in the PE group compared with that in the normal group, suggesting that the expression level of PLGF should increase in early and mid-pregnancy to reduce the incidence of PE. In the present study, the AUCs of AFP, PLGF, β-hCG and PAPP-A in predicting the development of PE were 0.618, 0.645, 0.690 and 0.645, respectively, with certain values in the diagnosis of PE. In addition, it was also found that the AUC of the combined detection of AFP, PLGF, β-hCG and PAPP-A was 0.834, and the prediction efficiency of the combined detection was significantly increased compared with that of each individual marker alone, which was helpful to the early detection and intervention of PE as well as the development of effective measures to reduce the incidence of PE.

### Limitations:

However, limitations may exist in the present study due to the small sample size, hence further studies with larger sample sizes are needed.

## CONCLUSIONS

Prenatal screening markers in early pregnancy combined with serum PLGF can predict the development of PE, and its diagnostic efficiency is higher than that of each individual marker alone, with a favorable application prospect.

### Authors’ Contributions:

**ZS:** Designed this study and prepared this manuscript, and was responsible and accountable for the accuracy or integrity of the work.

**WW:** Collected and analyzed clinical data, participated in acquisition, analysis, or interpretation of data.

All authors read and approved the final manuscript.
